# 

**Published:** 1840

**Authors:** 


### TO CORRESPONDENTS.

To the conductors of "the American Journal of Dental Science," it is always a source of pleasure to receive communications from their friends and patrons ; yet they would remind those writing to them, whether to either of the editors, any of the Publishing Committee, or Treasurer, that the postage should in all instances, except by agents enclosing remittances, be paid. To their correspondents individually, the amount is but small, but to the conductors of the Journal, it is a burthensome item, and when it is considered that their subscription is barely sufficient to defray the expenses of publication, their request cannot be regarded as unreasonable.

We are requested to state, that the Mr. Clark alluded to by Mr. Noyes, in his article on "Metallic Pastes and Amalgams," was not Mr. F. H. Clark, of Baltimore ; but Mr. A. Clark, of Pen Yan, whose inquiries had been given in a previous number of the Journal.

The collaborators of this Publication, in addition to the Editors and Publishing Committee, are,—

H. H. Hayden, M. D., Baltimore
J. F. Flagg, M. D., Boston
B. A. Rodriguez, M. D., Charles. S. C.
C. S. Brewster, Paris, France
Leonard Koecker, M. D. London, Eng.
Enoch Noyes, Baltimore,
Harvey Burdell, M. D. New-York.
J. P. Hullihen, M. D., Wheeling, Va.
Leonard Macall, M. D. Baltimore
E. Maynard, M. D., Washington City
James D. McCabe, Richmond, Va.
S. M. Sheppard, Petersburg, Va.
George Merryman, Baltimore.

### AGENTS FOR THE
### AMERICAN JOURNAL OF DENTAL SCIENCE.

Doct. W. N. McKenney, *Fredericksburg, Va.*

F. B. Chewning, *Richmond, Va.*

A. Pierce, *Sussex Court House, Va.*

Chas. S. Pleasants, *Painesville, Ohio.*

David Walker & Asahel Jones, 174 *Fulton Street, New York.*

Edward Taylor *Maysville K. Y.*

O. P. Laird, M. D., *Columbus, Ga.*

John D. Chevalier, 135 *Fulton-st. N. Y.*

Geo. Washington Parmly, *N. Orleans.*

A. W. Brown, *New-York.*

F. M. Boykin, M. D. *Smithfield, Va.*

G. McDonald, M. D. *Macon, Geo.*

E. Maynard, M. D. *Washington City.*

McAllister & McNaughton, *Albany.*

M. K. Bridges, *Brooklyn.*

Blanding & Avery, *Columbia, S. C.*

Eleazer Gidney, *England.*

S. Wheeler, M. D *Murfreesboro, N. C.*

W. H. H. Davis, M. D. *Cassville, Oneida Co. New-York.*

B. A. Rodriguez, M. D. *Charles. S. C.*

Ferguson & Goddard, *Louisville, Ky.*

L. S. Stringfellow, *Baltimore.*

J. P. Norman, *Little Rock, Arkansas.*

E. E. Crowfoot, *Middletown, Conn.*

Samuel Highley, *Fleet-st. London.*

